# The effect of gestational weight gain on perinatal outcomes among Chinese twin gestations based on Institute of Medicine guidelines

**DOI:** 10.1186/s12884-019-2411-7

**Published:** 2019-07-24

**Authors:** Dongxin Lin, Dazhi Fan, Shuzhen Wu, Gengdong Chen, Pengsheng Li, Huiting Ma, Shaoxin Ye, Jiaming Rao, Huishan Zhang, Meng Zeng, Yan Liu, Xiaoling Guo, Zhengping Liu

**Affiliations:** 1 Foshan Institute of Fetal Medicine, Southern Medical University Affiliated Maternal & Child Health Hospital of Foshan, 11 Renminxi Road, Foshan, 528000 Guangdong China; 2 Department of Obstetrics, Southern Medical University Affiliated Maternal & Child Health Hospital of Foshan, Foshan, 528000 Guangdong China

**Keywords:** Twin, Gestational weight gain, Preterm birth, Gestational hypertensive disorder, Pre-eclampsia, Birth weight

## Abstract

**Background:**

Gestational weight gain (GWG) has implications for perinatal outcomes, the guidelines for maternal weight gain, however, remain understudied among twin pregnancies. This study aimed to assess the associations between perinatal outcomes and GWG among twin pregnancies, based on the US institute of Medicine (IOM) 2009 guidelines.

**Methods:**

A retrospective cohort study of pregnant women with viable twins ≥26 weeks of gestation, was conducted in Foshan, China, during July 2015 and June 2018. Maternal BMI was categorized based on Chinese standard and GWG was categorized as below, within and above the IOM 2009 recommendations. Underweight women were excluded for analysis. Perinatal outcomes were compared among these groups. To assess the independent impact of GWG on the perinatal outcomes, conventional multivariable regression and general estimated equation (GEE) were utilized for maternal outcomes and neonatal outcomes, respectively.

**Results:**

A total of 645 mothers with twin pregnancies were included, of whom 15.0, 41.4 and 43.6% gained weight below, within and above guidelines, respectively. Compared to weight gain within guidelines, inadequate weight gain was associated with increased risks in spontaneous preterm birth < 37 weeks (aOR:3.55; 95% CI: 1.73–7.28) and < 35 weeks (aOR:2.63; 95% CI: 1.16–5.97). Women who gained weight above guidelines were more likely to have gestational hypertension disorder (aOR: 2.36; 95% CI: 1.32–4.21), pre-eclampsia (aOR: 2.59; 95% CI: 1.29–5.21) and have fetuses weighted >90th percentile and less likely to have fetuses weighted < 2500 g and < 1500 g.

**Conclusions:**

Maintenance of gestational weight gain within the normal range could decrease the risk of adverse perinatal outcomes. However, the causality between pre-eclampsia and gestational weight gain requires further investigations.

## Background

Twin gestations, different from singleton gestations, are associated with significant adverse perinatal outcomes, such as fetal growth restriction, intrauterine fetal death, preterm birth and perinatal mortality [1, 2]. To date, many efforts have been developed in identifying factors which may jeopardize maternal and fetal health.

As reported in previous studies, gestational weight gain (GWG) has implications for the health of mother and child. Women with insufficient weight gain during gestation were observed to be associated with lower birth weight whereas those with excessive weight gain were associated with macrosomia, postpartum weight retention and offspring obesity [3–6]. The demand for nutrition supply, theoretically, should be higher for twin pregnancies than singletons [7]. Guidelines of GWG for singleton pregnancies were well established, whereas those for twin pregnancies were undeveloped. In 2009, the US institute of Medicine (IOM) released revised recommendations on maternal GWG for twin gestations [8]. These recommendations represented the interquartile range of weight gain among women who deliver twins with a mean weight of ≥2500 g at term (37–42 weeks of gestation) and were referred as provisional.

One of the most important questions comes to their validity since there are a significant proportion of twins born before 37 weeks of gestation and other perinatal outcomes to be assessed. Multiple researches from western countries had attempted to examine these guidelines on various perinatal outcomes among twin pregnancies, however, these studies yielded inconsistent results possibly due to several limitations, such as small sample size, exclusion of monochorionic or pre-term pregnancies [9–13]. Besides, as the Chinese adult body mass index (BMI) category standards for normal weight, overweight and obesity were lower than WHO standards, it is not clear whether these guidelines could apply to Chinese women with twin gestations.

In the view of the ever-increasing incidence of twin births in China [14], it is imperative to provide more evidence regarding this issue, in order to optimize maternal and fetal outcomes. Therefore, this study aimed to elucidate the association between maternal GWG and a series of perinatal outcomes, based on 2009 IOM recommendations.

## Methods

### Study design

This retrospective cohort study was conducted between July 2015 and June 2018, in a tertiary maternal and child health hospital in Foshan, China. All obstetric records of eligible subjects were systematically reviewed, and relevant data were extracted by pre-designed standardized forms. The study was approved by the ethics committee of Southern Medical University Affiliated Maternal & Child Health Hospital of Foshan (ethics approval number: FSFY-20180903).

### Inclusion and exclusion criteria

All pregnant women who aged between 18 and 55 years, had first antenatal visit in the first trimester and gave birth to viable twins at gestational age of ≥26 weeks were eligible for inclusion. Exclusion criteria included congenital anomalies, twin-to-twin transfusion (TTTS), monoamniotic twins, intrauterine death. Women were also excluded if there is no available information on maternal height or weight at the first prenatal visit or delivery.

### Information collection

Demographic and obstetric information were collected including maternal age, educational level, chronic hypertension, pre-existing diabetes mellitus, parity, obstetric history, use of assisted reproductive technology (ART), cesarean section, chorionicity, gestational age at delivery, maternal BMI and maternal weight gain at delivery.

Gestational age at delivery was calculated by the last menstrual period and confirmed by early sonography. Since pre-gestational weight was not available in medical chart, we used the measured weight in the first antenatal visit before 14 weeks instead to determine maternal BMI and gestational weight gain, like prior studies [15–17]. Maternal BMI, calculated by dividing their weight (in kilograms) by height (in meters squared), was categorized as underweight (BMI < 18.5 kg/m^2^), normal weight (18.5–23.99 kg/m^2^), overweight (24–27.99 kg/m^2^) and obesity (≥28 kg/m^2^) according to the standard of the Working Group on Obesity in China [18]. Gestational weight gain (GWG) was assessed as previous studies [13, 19–22]. The rates of recommended weight gain, obtained by dividing the recommended weight gain by 37 weeks, were as follows: 0.459–0.676 kg per week for normal weight; 0.378–0.622 kg per week for overweight and 0.297–0.514 kg per week for obesity. GWG of subjects, calculated by dividing the total weight gain by gestational weeks from the initial antenatal visit, was compared against the rate of recommended weight gain and define as below, within and above the IOM guidelines. Due to no recommendations for underweight pregnant women with twin gestations, those with BMI < 18.5 kg/m^2^ were not included for analysis.

### Outcomes of interest

The primary outcome of the current study were preterm birth (PTB, including spontaneous and iatrogenic PTB) < 37 , < 35 and < 32 weeks and different phenotypes of spontaneous PTB. Other outcomes included gestational hypertensive disorder, gestational hypertension, pre-eclampsia, anemia, preterm premature rupture of membranes (PPROM), small for gestational age (SGA) <10th percentile, large for gestational age (LGA) >90th percentile, low birth weight (LBW) < 2500 g and < 1500 g, neonatal intensive care unit (NICU) admission and neonatal respiratory distress syndrome (NRDS). Incidence of gestational diabetes mellitus (GDM) was reported but not evaluated as an outcome in this study since nutritional counseling and dietary adjustment may occur and have a consequent impact on GWG after the diagnosis of GDM. Gestational hypertension was defined as a new development of a blood pressure of ≥140/90 mmHg after 20 weeks’ gestation without proteinuria. A diagnosis of pre-eclampsia (PE) was made when a blood pressure of ≥140/90 mmHg and proteinuria of ≥300 mg/24 h were simultaneously found. Gestational hypertensive disorder was defined when at least one of the following complications occurred: gestational hypertension, preeclampsia and eclampsia [23]. Gestational anemia was diagnosed if the hemoglobin concentration was below 11.0 g/dl before delivery. SGA and LGA were defined when the birth weight was below the 10th percentile and above the 90th percentile for gestation age and sex based on twin birth weight curves in Chinese twins, respectively [24, 25].

### Statistical analysis

All statistical analyses were performed using Stata, version 13.1. The baseline characteristics and outcomes were compared between three GWG categories. Continuous variables with approximately gaussian distribution were presented as mean ± SD and analyzed by analysis of variance (ANOVA). Categorical variables were presented as frequency and accompanying percentage and analyzed by chi-square test or Fisher’s exact test, when appropriate. We applied multivariable logistic regression models to estimate the independent influence of maternal weight gain on perinatal outcomes. Weight gain within IOM guidelines served as the reference. For neonatal outcomes, logistic models with general estimated equations (GEE) were used in order to address the correlation between fetuses in a paired set. A priori determined variables including maternal age, maternal BMI classification, chorionicity as well as gestational age at delivery. All the effect estimates were reported as adjusted odds ratios (aOR) and accompanying 95% confidence intervals (CI) and presented graphically as forest plots. All *P*-values were two tailed, and *P*-values < 0.05 were considered as statistical significance.

## Results

**Fig. 1:**
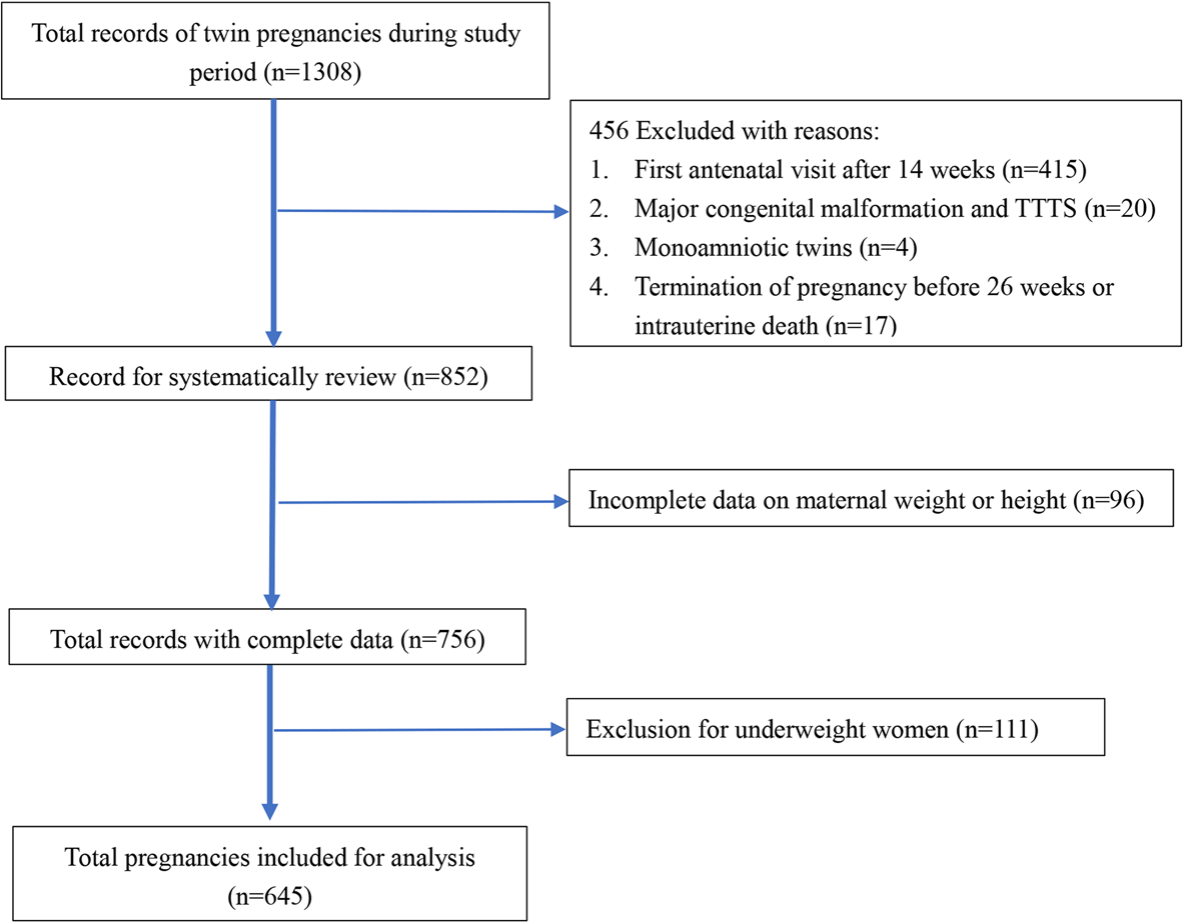
Flowchart of selection of eligible subjects

There were 1308 women with twin pregnancy admitted to maternal and child health hospital in Foshan, China, during the study period. Six-hundred and forty-five women with 1290 infants were finally included for analysis after excluding records of pregnancies which did not meet our inclusion criteria (*n* = 552), and underweight women (*n* = 111) (Fig. 1).

### Baseline characteristics

Characteristics of included twin pregnancies were presented in Table 1. Of included women, 15.04, 41.40 and 43.57% were below, within and above the recommended weight gain, respectively. The mean maternal age of eligible participants was 32.1 ± 4.3 years, and a significant difference was found between women with weight gain below, within and above IOM guidelines (*P* = 0.007). Approximately 15% of included pregnancies were monochorionic-diamniotic. There were no significant differences between the three groups for maternal BMI classification, nulliparity, chorionic hypertension, pre-existing diabetes mellitus, college degree, use of ART, cesarean section and prior cesarean section, spontaneous abortion and induced abortion. The incidence of gestational diabetes mellitus was significantly different across different GWG categories, highest among women with inadequate weight gain compared to other groups (*P* < 0.001).

**Table 1:** Characteristics of twin pregnancies by maternal pre-pregnancy BMI classification and by gestational weight gain according to Institute of Medicine Guidelines

Characteristics	All participants (*n* = 645)	Weight gain within IOM guideline (*n* = 267)	Weight gain below IOM guideline (*n* = 97)	Weight gain above IOM guideline (*n* = 281)	*P*-value
Maternal age, year	32.1 ± 4.3	32.7 ± 4.4	32.0 ± 4.3	31.5 ± 4.2	0.007
Maternal BMI classification					
Normal	475 (73.64)	190 (71.16)	69 (71.13)	216 (76.87)	0.506
Overweight	130 (20.16)	58 (21.72)	23 (23.71)	49 (17.44)	
Obesity	40 (6.2)	19 (7.12)	5 (5.15)	16 (5.69)	
Nulliparity	415 (64.34)	159 (59.55)	66 (68.04)	190 (67.62)	0.102
Chorionic hypertension	2 (0.31)	1 (0.37)	1 (1.03)	0 (0)	0.147
Pre-existing diabetes mellitus	11 (1.78)	4 (1.57)	4 (4.26)	3 (1.12)	0.131
College degree^a^	283 (49.13)	126 (53.85)	44 (51.76)	113 (43.97)	0.080
Use of ART	486 (75.35)	203 (76.03)	74 (76.29)	209 (74.38)	0.880
Prior cesarean section	99 (15.35)	39 (14.61)	13 (13.4)	47 (16.73)	0.668
Prior spontaneous abortion	131 (20.31)	65 (24.34)	15 (15.46)	51 (18.15)	0.086
Prior induced abortion	137 (21.24)	58 (21.72)	21 (21.65)	58 (20.64)	0.948
MCDA twin gestation	97 (15.04)	35 (13.11)	14 (14.43)	48 (17.08)	0.422
Gestational diabetes mellitus	154 (23.88)	69 (25.84)	40 (41.24)	45 (16.01)	< 0.001
Cesarean section	635 (98.45)	263 (98.50)	96 (98.97)	276 (98.22)	1.000

^a^missing data on 69 cases

### Maternal outcomes according to different IOM classification of GWG

The maternal outcomes were compared among women with inadequate, adequate and excessive GWG and presented in Table 2. Both gestational hypertensive disorder and pre-eclampsia, were significantly different among these groups (*P* = 0.004 and *P* = 0.006, respectively). On the contrary, gestational hypertension did not differ among these study groups (*P* = 0.643). None of the included patients had eclampsia. A higher rate of preterm birth < 35 weeks was found among women with inadequate weight gain (24.74%) compared to those with adequate (18.35%) and excessive weight gain (12.81%) (*P* = 0.018). No difference was observed in preterm birth < 37 and < 32 weeks (*P* = 0.422 and *P* = 0.692, respectively). When we analyzed spontaneous preterm birth, we found increases in spontaneous PTB < 37 and < 35 weeks in inadequate weight gain group compared to other groups (*P* < 0.001 and *P* = 0.001, respectively). The incidence of spontaneous PTB < 32 weeks was highest in inadequate weight gain group although the difference was near to statistical significance (*P* = 0.056). There were no significant differences in gestational anemia and PPROM between the three groups (all *P*-value > 0.05).

**Table 2:** Maternal outcomes by maternal pre-pregnancy BMI classification and by gestational weight gain according to Institute of Medicine Guidelines

Characteristics	All participants (*n* = 645)	Weight gain within IOM guideline (*n* = 267)	Weight gain below IOM guideline (*n* = 97)	Weight gain above IOM guideline (*n* = 281)	*P*-value
Gestational hypertensive disorder	65 (10.08)	19 (7.12)	5 (5.15)	41 (14.59)	0.004
Gestational hypertension	20 (3.1)	7 (2.62)	2 (2.06)	11 (3.91)	0.643
Pre-eclampsia	45 (6.98)	12 (4.49)	3 (3.09)	30 (10.68)	0.006
Gestational anemia	273 (42.33)	101 (37.83)	49 (50.52)	123 (43.77)	0.077
Preterm premature rupture of membrane	76 (11.78)	37 (13.86)	12 (12.37)	27 (9.61)	0.299
Preterm birth < 37 weeks	472 (73.18)	189 (70.79)	75 (77.32)	208 (74.02)	0.422
Preterm birth < 35 weeks	109 (16.9)	49 (18.35)	24 (24.74)	36 (12.81)	0.018
Preterm birth < 32 weeks	27 (4.19)	12 (4.49)	5 (5.15)	10 (3.56)	0.692
Spontaneous Preterm birth < 37 weeks	50 (7.75)	17 (6.37)	18 (18.56)	15 (5.34)	< 0.001
Spontaneous Preterm birth < 35 weeks	34 (5.27)	14 (5.24)	12 (12.37)	8 (2.85)	0.001
Spontaneous Preterm birth < 32 weeks	13 (2.02)	5 (1.87)	5 (5.15)	3 (1.07)	0.056

**Fig. 2:**
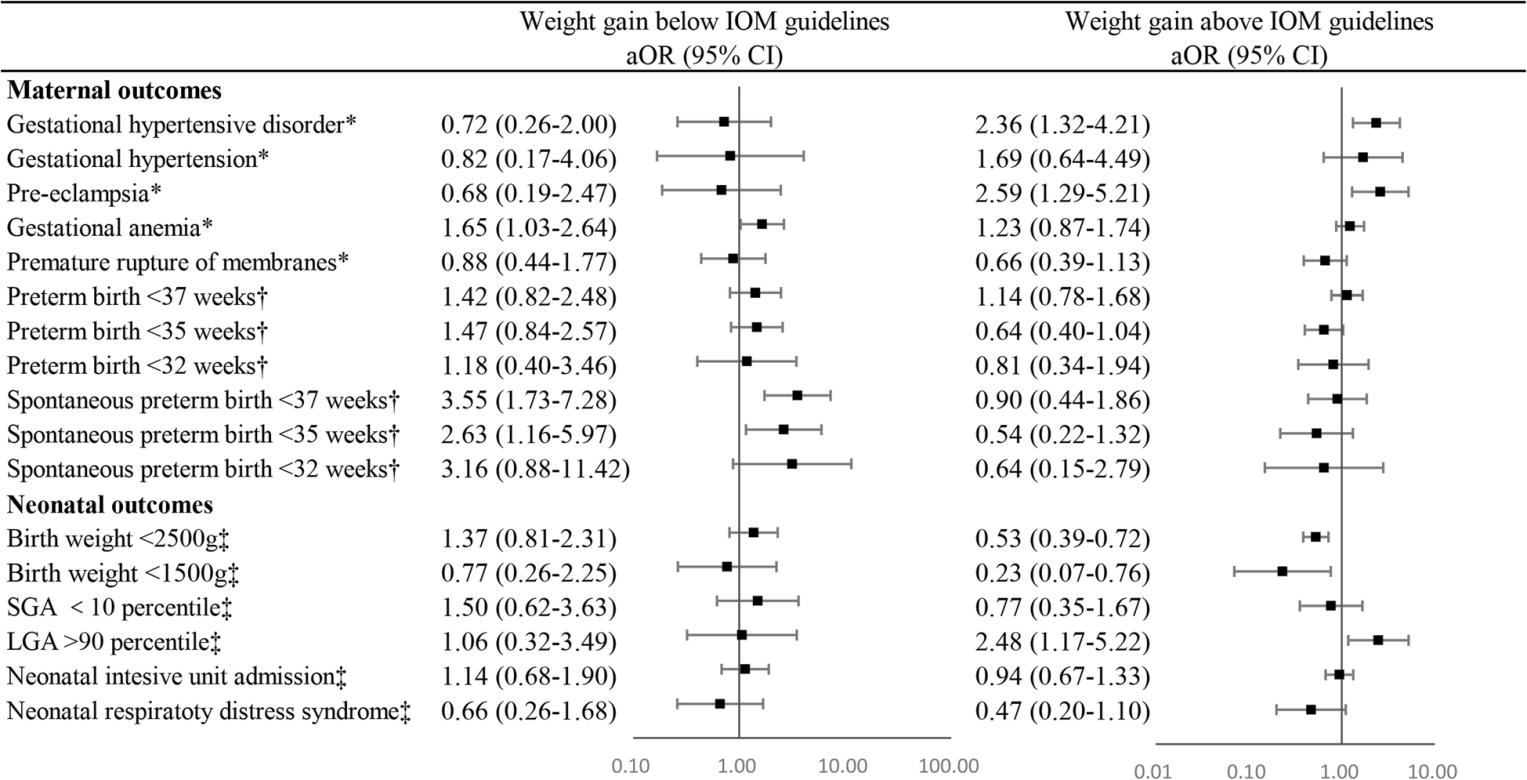
Multivariable logistic analysis of associations between perinatal outcomes and GWG according to 2009 IOM guidelines. GWG, gestational weight gain; aOR, adjusted odds ratio; CI, confidence interval; SGA, small for gestation; LGA, large for gestation; *, adjusted for maternal age and maternal BMI classification; †, adjusted for maternal age, maternal BMI classification and chorionicity; ‡, GEE were utilized and adjusted for maternal age, maternal BMI classification, chorionicity and gestational age at delivery

After we controlled for potential confounders in multivariable logistic regression models, as shown in Fig. 2, neither inadequate nor excessive GWG was associated with preterm birth < 37, < 35 and < 32 weeks. Nevertheless, higher risks of spontaneous PTB < 37 and < 35 weeks were observed among women who gained weight below the recommendations compared to normal weight gain women (adjusted OR of 3.55 [95% CI: 1.73–7.28] and 2.63 [95% CI: 1.16–5.97] for spontaneous PTB < 37 and < 35 weeks, respectively). Women with inadequate weight gain also had higher odds of gestational anemia (adjusted OR: 1.65; 95% CI: 1.03–2.64). Women who gained weight above IOM guidelines had 136% higher odds of gestational hypertensive disorder (95% CI: 1.32–4.21) and 159% higher odds of pre-eclampsia (95% CI: 1.29–5.21) compared to women who gained weight within the guidelines (Fig. 2).

### Neonatal outcomes according to different IOM classification of GWG

The current study included a total of 1290 fetuses in the analysis. Results of univariable analysis were shown in Table 3. Statistically significant differences were found in birth weight < 2500 g and < 1500 g, LGA, NICU admission and NRDS between women who gain weight under, within and above recommended GWG (all *P*-value < 0.05).

**Table 3:** Neonatal outcomes by maternal pre-pregnancy BMI classification and by gestational weight gain according to Institute of Medicine Guidelines

Characteristics	All fetuses (*n* = 1290)	Weight gain within IOM guideline (*n* = 534)	Weight gain below IOM guideline (*n* = 194)	Weight gain above IOM guideline (*n* = 562)	*P*-value
Birth weight < 2500 g	823 (63.8)	360 (67.42)	149 (76.8)	314 (55.87)	0.003
Birth weight < 1500 g	58 (4.5)	32 (5.99)	11 (5.67)	15 (2.67)	0.004
Small for gestational age	69 (5.35)	29 (5.43)	16 (8.25)	24 (4.27)	0.109
Large for gestational age	71 (5.5)	19 (3.56)	7 (3.61)	45 (8.01)	0.003
Neonatal intensive unit admission	490 (37.98)	206 (38.58)	88 (45.36)	196 (34.88)	0.004
Neonatal respiratory distress syndrome	79 (6.12)	44 (8.24)	13 (6.70)	22 (3.91)	0.009

Results of general estimated equations reported no significant associations between inadequate weight gain and adverse outcomes in our study after adjusting for maternal age, maternal BMI classification, chorionicity and gestational age at delivery. Nevertheless, excessive weight gain increased the risk of LGA (adjusted OR: 2.48; 95% CI: 1.17–5.22) and also decreased the risks of birth weight < 2500 g (adjusted OR: 0.53; 95% CI: 0.39–0.72) and < 1500 g (adjusted OR: 0.23; 95% CI: 0.07–0.76). (Fig. 2).

## Discussion

This study sought to evaluate the associations between gestational weight gain and perinatal outcomes among Chinese twin gestations, according to 2009 IOM guidelines. We found that, on one hand, weight gain below the IOM guidelines was associated with significant increases in spontaneous preterm birth < 37 and < 35 weeks as well as anemia; on the other hand, weight gain above the guidelines was associated with higher rates of gestational hypertensive disorder, pre-eclampsia and fetal macrosomia and lower rates of birth weight < 2500 g and < 1500 g.

Preterm birth, the primary outcomes in the current study, predominates in adverse outcomes among twin pregnancies. Identifying indicators of PTB would be beneficial for optimization of neonatal outcomes. We found none of the association between maternal weight gain and PTB < 37, < 35 or < 32 weeks. The increased risks of spontaneous PTB < 37 and < 35 weeks, nevertheless, were found among women with weight gain below the recommended level, compared to those with weight gain within the recommended range. These results were similar with the previous findings of González-Quintero et al. They reported women with twin gestations who had weight gain at or above guidelines, could had a significantly lower rate of spontaneous preterm birth than those who had weight gain below the guidelines [21]. Rather than combining women with weight gain at or above the guidelines as a reference, we did only use normal weight gain as the reference, compared to previous studies [21, 22]. Thus, our results may provide more detailed information on the association between maternal weight gain and spontaneous preterm birth. Algeri et al. [11] reported that excessive GWG is associated with higher risk of spontaneous preterm delivery < 37 weeks as well. This finding is less valid due to the limited number of women with inadequate weight gain. The mechanism how maternal weight gain influence the spontaneous preterm birth is uncertain. Similar incidence of PPROM was observed between three GWG categories in our results, which was supported by a former study [9]. In addition, an increased risk of gestational anemia was observed among women with inadequate weight gain. Recently Pécheux et al. [26] speculated that strengthening uterine contractions may occur by a surge of stress hormones, induced by placenta dysfunction in the anemia status. Based on our results, we stressed the necessity of sufficient weight gain among women with twin gestations.

The association between weight gain above recommended GWG and gestational hypertensive disorder reported in our study was endorsed by those of previous studies [10, 11, 27]. Interestingly, when gestational hypertension and pre-eclampsia were analyzed separately, we found an association between excessive weight gain and pre-eclampsia but not gestational hypertension. Existing evidence was controversial on the correlation between maternal weight gain and these vascular complications during twin pregnancy. Some studies reported nonsignificant difference of pre-eclampsia among different GWG categories [9, 12, 28]. The study of Lal et al. [20] showed that the association between preeclampsia and excessive GWG only found in the normal-weight/underweight group but not in overweight and obese groups. The conflicting results, which could be due to the heterogeneity of the study population and sample size, made it difficult to draw a conclusion on whether these weight gain thresholds may be beneficial in decreasing the risk of these complications. Kosinska-Kaczynska et al. [9] suggested that IOM guidelines could be unsuitable for analyzing pre-eclampsia among twin pregnancies, as they found no overlaps between the GWG ratio range associated with pre-eclampsia and specific IOM-recommended GWG value. Our results may imply the reverse causality between excessive weight gain and pre-eclampsia, since women who have pre-eclampsia develop edema more frequently than those who solely have gestational hypertension. Further prospective studies considering edema as a covariate are warranted to elucidate the causality between weight gain and pregnancy-related hypertensive disorder.

A few of previous studies evaluated the association between maternal weight gain and birth weight without including the length of gestation or chorionicity as confounders [20, 21, 27–29]. We thought that these variables play significant roles in fetal growth among twins thus we adjusted these confounders for neonatal outcomes. We found no associations between weight gain below IOM guidelines and neonatal outcomes although higher rates of birth weight < 2500 g and SGA were observed in the inadequate weight gain group. In this study, maternal BMI was categorized based on Chinese adult standard, different from those in previous studies conducted in western countries [9, 13, 21, 22]. These results raised a question whether the lower limit of IOM recommended weight gain has enough sensitivity to detect the SGA twins among Chinese pregnant women, which deserves further study. Alternatively, the lack of significant association could be partly attributed to the smaller number of women gaining weight inadequately than other groups. Large studies suggested that GWG below the IOM recommendations was associated with an increased risk of SGA twins [27, 29]. On the opposite, we found that weight gain above the recommended GWG was associated with a higher risk of LGA, which is consistent with the previous finding of Ozcan et al. [27]. In addition, this GWG category also decreased the risk of birth weight < 2500 g and < 1500 g.

As gestational weight gain is a satisfying index for evaluating maternal nutrition and a modifiable factor during pregnancy, our results are of clinical significance for patients and clinicians to achieve improved pregnancy outcomes among twin gestations. IOM recommendations could provide a good reference in managing weight gain for Chinese women with twin pregnancies, despite of difference in BMI category between Chinese standard and WHO standard. Furthermore, an intervention on maternal nutrition and weight gain could help to reduce the risk of the above-mentioned adverse outcomes.

Since the 2009 IOM recommendations are for the general twins, we included both monochorionic diamniotic and dichorionic diamniotic twins. Therefore, our study possesses more profound significance than those which only included dichorionic twins [30, 31] or did not have information on chorionicity [27, 29]. In addition, different from majority of prior studies [10, 16, 20, 28], one merit of this study was to apply GEE models to assess neonatal outcomes, which could yield more robust and valid effect estimates and confidence intervals with the correlation in a twin set being controlled. Nevertheless, we also acknowledged some limitations in our study. Firstly, the inherent potential for bias unable to be avoided in the retrospective design may impede the interpretation of the causal relationship between maternal weight gain and pregnancy outcomes. Secondly, due to the limited number in twin pregnancies with weight gain below recommended GWG group, some outcomes need more assessment in further studies. Thirdly, since pre-pregnancy weight was not available in the medical charts, we used the measured weight at the first prenatal visit before 14 weeks to calculate the maternal BMI and maternal weight gain. This value has some limitations, despite its satisfying correlation with pre-pregnancy weight [32].

## Conclusions

Weight gain below the provisional guidelines for twins is associated twin spontaneous preterm birth while weight gain above the guidelines is associated with gestational hypertensive disorder, pre-eclampsia and LGA. Further prospective research is warranted to confirm the causality of these associations.

## Data Availability

The datasets used or analyzed in current study are available from the corresponding author on reasonable requests.

## Abbreviations

ANOVA: Analysis of variance
aOR: adjusted odds ratio
CI: Confidence interval
ART: Assisted reproductive technology
BMI: Body mass index
GDM: Gestational diabetes mellitus
GEE: General estimated equation
GWG: Gestational weight gain
IOM: Institute of Medicine
LBW: Low birth weight
NICU: Neonatal intensive care unit
NRDS: Neonatal respiratory distress syndrome
PPROM: Preterm premature rupture of membranes
PTB: Preterm birth
SGA: Small for gestational age
TTTS: Twin-to-twin transfusion
